# Editorial

**Published:** 1849-09

**Authors:** 


### THE MEDICAL EXAMINER.

PHILADELPHIA, SEPTEMBER, 1849.

### DISAPPEARANCE OF THE CHOLERA FROM PHILADELPHIA.

*Health Office, Philadelphia.*—At a meeting of the Board of Health, held to-day, [Aug. 20,] the following preamble and resolution were adopted:

Whereas, in the opinion of the Board, Cholera no longer exists in the community as an epidemic; therefore,

Resolved, That "Daily Bulletins" will no longer be issued by this Board, unless our city and county should be visited by a return of "epidemic cholera." By order of the Board.

SAMUEL P. MARKS, Clerk.

Under the authority of the above official notice, we are happy to announce the entire disappearance of the Epidemic Cholera from our midst. The citizens of Philadelphia have great reason to congratulate themselves that they have been so lightly visited. We believe this exemption to be owing in a great measure, under Providence, to the energetic and unceasing efforts of our Sanatory Boards in providing and maintaining guard against the inroads of the pestilence, faithfully supported and assisted as they have been by the municipal authorities and the medical profession.

To the last it is especially a subject of rejoicing, that, for a season at least, they may rest from their labors. During the time of trial among us they have been "instant in season and out of season," ready at all times, without thought of self, to fulfil the unusually arduous duties of their calling.

Circumstances beyond our control have prevented us from presenting a comparison of this epidemic with that of 1832. We hope to do so in the October number.

### ST. JOSEPH'S HOSPITAL.

An admirable charity, having the above title, has recently been chartered, and has commenced operations in a most eligible locality in the vicinity of the city.

The Hospital has been placed in charge of the religious order of the *Sisters of St. Joseph*, who by their vows make attendance upon the sick a part of their duty.

Although under the auspices of one religious denomination, this institution does not limit its good work to that communion alone, but as its charter indicates, "its benefits and advantages shall be extended to the sick, without reference to *creed, country or color.*" It is contemplated that the buildings when completed will accommodate one hundred and twenty patients, who, for the moderate sum of three dollars per week will obtain all the comforts needed by the sick, to be obtained in private houses at five. For every five of these pay patients, it is proposed to receive one beneficiary.

The medical staff, comprising some of our most eminent physicians, are as follows: W. E. Horner, M. D., Samuel Jackson, M. D., Alfred Stillé, M. D., W. V. Keating, M. D.

---

To the Editors of the Medical Examiner.

GENTLEMEN:—While many others are engaged in inventing new theories or reviving old remedies for Cholera—in describing the wonderful phantasmagoria of Microscopic Anatomy, or roaming through the Elysian fields of Anæsthetic Medicine, allow me to solicit a little information, on a smaller matter, through your valuable journal.

You, or some other member of the profession, can confer an important favor on many of us, by informing us how we may preserve our gum elastic catheters and bougies from the influence of the warm summer weather. I have been buying gum elastic catheters and bougies occasionally for the last twelve years, and have seldom found it possible to preserve them fit for use through the following summer.

As soon as the weather grows warm the elastic gum coating begins to soften and they adhere to each other, to the side of the case, or to whatever other substance they may be enveloped in. When separated by force, the polished surface is destroyed and the catheter or bougie left entirely unfit for use. If a number of them be left lying in contact, they become glued together so firmly that it is impossible to separate them without tearing the gum coating off. If kept separate, they soften and adhere to anything that I have been able to place them in contact with, in defiance of oils or unguents. Whether the fault is in the particular manufacture I have received, or whether all are liable to the same objection, I am unable to say. Out of about three dozen catheters and bougies purchased at different times and from different druggists, I have not one now fit for use, though I have never made any use whatever of two-thirds of them, and have tried diligently to preserve them. A friend of mine succeeds by keeping them in a cool cellar, but many of us have no cellars.

This is a source of much inconvenience to me, as well as other country practitioners, as the diseases of the urinary organs requiring their use are of a most painful and troublesome character, and not of unfrequent occurrence. Could not some improvement be made in them that would obviate the difficulty? Or, if not, could not the gutta percha, or some other article, be substituted for the gum elastic?

Yours respectfully,

C. J. Clark, M. D.

*Jacksonville, Ala.*, July 22, 1849.

[In reply to our correspondent, we can only say that the same difficulty has occurred to practitioners in this section of country. To a certain extent it may be remedied by keeping the catheters and bougies in an air-tight tin box, having previously dusted them over with finely-powdered starch, (ordinary hair powder,) which can readily be wiped off before using them.

It is doubtful whether the gutta percha could be substituted for the gum elastic, for although flexible, it does not possess sufficient elasticity for the purpose proposed. It might, perhaps, be advantageously combined with caoutchouc. We would be pleased to hear from any of our readers who can inform us further on this subject.—Eds. Ex.]

---

### ARMY MEDICAL DEPARTMENT.

On the 15th of October a Medical Board will assemble in Philadelphia, to examine candidates for the post of Assistant Surgeon in the army, of which there are several vacancies at present, as well as to provide for filling such vacancies as may occur during the year. The Board will continue in session two weeks. Applications for the post, or for permission to appear before the board, must be made to the Secretary of War, accompanied by testimonials as to moral character. Candidates must be between twenty-one and twenty-eight years of age.

---

### PENNSYLVANIA HOSPITAL.

Dr. C. D. Meigs has resigned the post of Physician to the Lying-in Department in this Institution, and Dr. Joseph Carson has been elected in his stead.

Dr. Carson brings to the appointment a large fund of experience, having been long and successfully engaged in this branch of his profession. We rejoice that the loss of Dr. Meigs' valuable services has been so well met in the election of his successor.

CHOLERA—DR. BIRD AND HIS SULPHUR PILLS.

It appears that Dr. Bird is about to distinguish himself still further, in his much vaunted specific—sulphur.

His pills, it seems, have shown themselves a trifle too potent, having produced in a young lady, at Detroit, in addition to the arrest of the diarrhœa, unmistakeable narcotism—an effect hitherto, we believe, never attributed to sulphur.

The Detroit Free Press, for June 16, contains the following curious statement, by Dr. Z. Pitcher, respecting the Chicago Remedy:

"I was called," says Dr. P., "to see a young lady, quite indisposed, whose symptoms were so analogous to those produced by a full dose of opium or morphine, that the inquiry was at once made of her friends whether she had taken a narcotic in any form, within the last few hours. Both by them and herself I was assured, that the only medicine she had taken in several days was one of Dr. Bird's sulphur pills, the evening before, to arrest a diarrhœa, which it had done very effectually.

"I am so strongly impressed with the idea that the symptoms of narcosis were occasioned by the pill, that I have obtained two others from the same source."

These last two, unfortunately for Dr. Bird and the sulphur, were submitted to A. R. Terry, M. D., who, upon chemical analysis, says he discovered *a notable portion of morphia*, or one of its salts! Dr. Terry, not satisfied with this, went still further, and "took," says he, "the pains to trace these pills, and have the strongest reasons to suppose—nay, I am sure—that they came from Dr. Bird, of Chicago, himself."—*Western Journal of Medicine and Surgery.*

